# MicroRNA-182 Regulates Neurite Outgrowth Involving the PTEN/AKT Pathway

**DOI:** 10.3389/fncel.2017.00096

**Published:** 2017-04-10

**Authors:** Wu M. Wang, Gang Lu, Xian W. Su, Hao Lyu, Wai S. Poon

**Affiliations:** Division of Neurosurgery, Department of Surgery, Prince of Wales HospitalThe Chinese University of Hong Kong, Hong Kong, China

**Keywords:** microRNA-182, axon outgrowth, dendrite, AKT, BCAT2

## Abstract

MicroRNAs are implicated in neuronal development and maturation. Neuronal maturation, including axon outgrowth and dendrite tree formation, is regulated by complex mechanisms and related to several neurodevelopmental disorders. We demonstrated that one neuron-enriched microRNA, microRNA-182 (miR-182), played a significant role in regulating neuronal axon outgrowth and dendrite tree formation. Overexpression of miR-182 promoted axon outgrowth and complexity of the dendrite tree while also increasing the expression of neurofilament-M and neurofilament-L, which provide structural support for neurite outgrowth. However, a reduction of miR-182 inhibited neurite outgrowth. Furthermore, we showed that miR-182 activated the AKT pathway by increasing AKT phosphorylation on S473 and T308 and inhibiting PTEN activity by increasing phosphorylation on S380. Inhibition of AKT activity with the PI3-K inhibitor LY294002 could downregulate AKT and PTEN phosphorylation and suppress axon outgrowth. In addition, we showed that *BCAT2* might be the target of miR-182 that takes part in the regulation of neuronal maturation; blockage of endogenous BCAT2 promotes axon outgrowth and AKT activity. These observations indicate that miR-182 regulates axon outgrowth and dendrite maturation involving activation of the PTEN/AKT pathway.

## Introduction

During brain development, neurons are generated in the ventricular zone and subventricular zone (SVZ) (Dehay and Kennedy, 2007). Neuronal maturation passes through five stages morphologically, including filopodia, immature neurites, axon outgrowth, mature neurites, and premature dendritic spines. After being dissociated from the embryonic brain, neurons form several filopodia; several hours later, the neurons generate numbers of immature neurites; one of these immature neurites extends rapidly and develops to axon, and other neurites become immature dendrites. When axons and dendrites are mature, the neurons form synaptic contacts which enable to transmit electrical signal (Arimura and Kaibuchi, 2007). During the neuronal maturation, large numbers of microRNAs contribute to these processes (Kosik, 2006).

MicroRNAs are a class of conserved non-coding RNAs containing about 22 nucleotides that modulate gene expression by targeting messenger RNA (mRNA), which leads to reduced translational efficiency, thereby influencing many biological processes. MicroRNAs are well-known to take part in many cellular processes, and have been found with distinct expression patterns in neural cells (Kosik, 2006). For example, miR-137 is specifically expressed in neurons compared with neural stem cells (NSCs) and astrocytes (Smrt et al., 2010). MicroRNAs also have different expression pattern in axon and dendrites. MiR-9 is expressed in axons of primary cortical neurons (Dajas-Bailador et al., 2012); miR-138, which functions to control dendrite development, is highly enriched in brain and localized within dendrites (Siegel et al., 2009).

Furthermore, microRNAs have also been demonstrated to take part in the regulation of neurite outgrowth and spine morphogenesis. Overexpression of miR-34a significantly decreases the number of neurite branches (Agostini et al., 2011), and miR-9 negatively regulates axon branching by targeting microtubule stability-related genes (Dajas-Bailador et al., 2012). MiR-134, a brain-specific microRNA, negatively modulates the size of dendritic spine of rat hippocampal neurons (Schratt et al., 2006). The miR-182/183/96 cluster is specifically expressed in sensory neurons and is involved in maintaining cone photoreceptor outer segments (Xu et al., 2007; Busskamp et al., 2014). MiR-182 has been recently found to prevent retinal degeneration (Lumayag et al., 2013). Quantitative real-time PCR and *in situ* hybridization reveal that the miR-182/183/96 cluster is highly expressed in dorsal root ganglion neurons, and the expression is decreased in injured neurons compared with controls (Aldrich et al., 2009). So far, there are no direct evidence for miR-182 regulating neurite growth in neurons of the central nervous system, but recent literatures identify that miR-182 is an important modulator of memory formation and regulates dendrite branching out of trigeminal sensory neurons (Griggs et al., 2013; Wang et al., 2016; Woldemichael et al., 2016).

MicroRNAs are involved in crucial biological processes by modulating signal transduction pathway (Inui et al., 2010). The PTEN/AKT pathway regulated by microRNAs plays important roles in neuronal maturation. MiR-9 and miR-124 regulate dendritic branching via AKT/GSK3β pathway (Xue et al., 2016); PTEN/miR-29a pathway modulates neurite outgrowth (Zou et al., 2015). In neuronal regeneration, PTEN/AKT pathway regulated by microRNA *bantam* enhances the regeneration of sensory neuron axons and dendrites (Song et al., 2012). Co-deletion of *PTEN* and *SOCS3* induces regrowth of retinal axons (Bei et al., 2016); both *PTEN* and *SOCS3* deletion greatly increases the intrinsic regenerative ability of injured retinal ganglion cells (RGCs), resulting in robust long-distance axon regeneration in optic nerve injury model (Sun et al., 2011).

In the cellular signal pathway, some essential genes are identified as downstream or upstream signals of PTEN/AKT. In the brain, branched-chain aminotransferase (BCAT) is a critical enzyme in the catabolism of the essential branched chain amino acids (BCAAs) leucine, valine, and isoleucine. In this catabolism, glutamate as a product of the BCAA catabolism is the major excitatory neurotransmitter and precursor of γ-aminobutyric acid (GABA). There are two BCAT isoforms, mitochondrial BCATm and cytosolic BCATc which are expressed in cultured astrocytes and neurons (Bixel et al., 2001; Castellano et al., 2007; Cole et al., 2012). BCAT2 is a kind of BCATm, which are ubiquitously presented in all tissues in the mitochondria of cells (Hull et al., 2012). It is a newly identified target of miR-182 that negatively regulates AKT activity, and BCAT2 depletion results in a significant increase in cardiomyocyte size and phosphorylation of AKT (S473) (Li et al., 2016). In our study, we investigated the functions of miR-182 in axon outgrowth and dendrite branching out of cortical neurons, and demonstrated that BCAT2/PTEN/AKT pathway might participate in the regulation of neuron maturation.

## Materials and Methods

### Ethics Statement

All of our experiments were performed in accordance with the recommendations of the Animal Experimentation Ethics Committee of The Chinese University of Hong Kong. The protocol was approved by the Animal Experimentation Ethics Committee of The Chinese University of Hong Kong (Ref. No. 16-060-MIS).

### Collection of Primary NSCs

Mouse NSCs were obtained from the SVZ of an adult mouse brain (Walker and Kempermann, 2014). Briefly, the lateral wall of lateral ventricle was dissected and dissociated into single cells by 0.05% trypsin-EDTA, and the cells were seeded into Petri dishes with KnockOut TM D-MEM/F-12 medium containing StemPro Neural Supplement (2%), bFGF (20 ng/ml), EGF (20 ng/ml), GlutaMAX-I Supplement (2 mM), heparin (6 units/ml) and ascorbic acid (200 μM). The SVZ-derived neurospheres were incubated for 6–7 days and replaced with fresh medium every 3 days. For neuron differentiation, NSCs were plated into dishes coated with poly-D-lysine (Sigma) and laminin (Invitrogen) in a 1:1 mix of Neurobasal Medium and DMEM/F12 supplemented with N2 (Gibco), B27 (Gibco), 10 ng/ml BDNF (Peprotech), and 200 mM ascorbic acid (Sigma). Half of the medium was replaced every other day. After 2 weeks of culture, the ratio of the mixture of Neurobasal Medium and DMEM/F12 was changed to 3:1; the N2 supplement was reduced to 0.5%, and the BDNF was increased to 20 ng/ml (Thier et al., 2012).

### Cortical Neuron Culture

Primary cortical neuron cultures were prepared from embryonic day 18.5 (E18.5) mouse brain. Chamber slides (Nunc) were coated with 100 μg/ml poly-L-lysine (Sigma) and 5 μg/ml laminin (Invitrogen) at 37°C in an incubator for 3 h to overnight, then washed twice with distilled water, and air dried 20 min. Cortices were digested with 1× trypsin-EDTA for 15 min at 37°C, and then the reaction was stopped with trypsin inhibitor for 3 min at room temperature. After washing with dissection buffer containing 1× HBSS without Ca^2+^ and Mg^2+^ (Invitrogen), 10 mM HEPES buffer (Invitrogen), 0.5% glucose and 100 units/ml antibiotics (penicillin and streptomycin) (Invitrogen), the tissues were triturated by gently pipetting in plating medium containing MEM without glutamine (Life Technologies), 10% FBS (Gibco), 1 mM L-glutamine (Invitrogen), 10 mM Hepes (Invitrogen), and 50 units/ml antibiotics (penicillin and streptomycin) (Invitrogen) until fully dissociated. Cells were diluted to an appropriate concentration and plated in chamber slides (Nunc) pre-coated with poly-L-lysine (Sigma) and laminin (Invitrogen). Three hours later, cells were grown in culture medium containing neurobasal medium (Invitrogen), 2% B27 supplement (Invitrogen), 0.5 mM L-glutamine (Invitrogen), and 50 units/ml antibiotics (penicillin and streptomycin) (Invitrogen) (Kaech and Banker, 2006).

### Western Blot

Proteins were extracted with RIPA buffer containing protease inhibitor cocktail, and the concentration of which was measured with a BCA kit. Proteins (10–20 μg) were loaded into 8–10% SDS-polyacrylamide gel. Following SDS-PAGE, proteins were transferred to nitroate cellulose membrane, blocked with 5% non-fat milk, incubated overnight with primary antibodies at 4°C, and washed three times with TBST for 10 min. After the proteins had been incubated with secondary antibodies for 2 h at room temperature, signals were detected by enhanced chemiluminescent (Thermo Fisher Scientific). The following primary antibodies were used: rabbit anti-PTEN (1:1000; Cell Signaling Technology #9188), rabbit anti-p^(Ser380)^ PTEN (1:1000; Cell Signaling Technology #9551), rabbit anti-AKT (1:1000; Cell Signaling Technology #4691), rabbit anti-p^(Ser473)^-AKT (1:1000; Cell Signaling Technology #4060), rabbit anti-p^(Thr308)^-AKT (1:1000; Cell Signaling Technology #13038), phospho-p44/42 MAPK (Erk1/2)^(Thr202/Tyr204)^ (1:1000; Cell Signaling Technology #9106), p44/42 MAPK (Erk1/2) (1:1000; Cell Signaling Technology #9102), mouse anti-β-actin (1:2000; ImmunoWay #YM3028), rabbit anti-Neurofilament-L (NF-L; 1:1000; Cell Signaling Technology #2837), mouse anti-Neurofilament-M (NF-M; 1:1000; Cell Signaling Technology #2838) and rabbit anti-BCAT2 (1:500; Abcam #ab95976). The secondary antibodies used were: anti-mouse HRP-linked antibody (1:2000; Cell Signal Technologies #7076) and anti-rabbit HRP-linked antibody (1:2000; Cell Signal Technologies #7074).

### Morphological Analysis

Cortical neurons were transfected with 60 nM scramble mimics as control, 60 nM miR-182-5p mimics, 60 nM negative control inhibitor mimics, 60 nM miR-182 inhibitor mimics, 60 nM negative control siRNA, 60 nM BCAT2 siRNA-1 mimics (TechDragon #siRNA no. 1330304) and 60 nM BCAT2 siRNA-2 mimics (TechDragon #siRNA no. 1330305) plus *GFP*-encoding plasmid at 1 day *in vitro* (DIV) by using Lipofectamine 3000 reagent (Invitrogen), incubated with lipofectamine 3000 and MicroRNA agomir or antagomir at 37°C, 5% CO_2_ for 24 h, washed once with 1× PBS and replaced with fresh medium. 36 h later, analyzed for axon outgrowth; also transfected at 5 DIV, and 48 h later, analyzed for dendrite development. The *GFP*-encoding plasmid is promoted by the CAG promoter, and the plasmid was a generous gift from Professor Zhao Hui (School of Biomedical Sciences, The Chinese University of Hong Kong).

Lengths of axons and dendrites were quantified by NIH Image J. The statistical analysis of total dendritic branch tip number (TDBTN), average dendrite branching length (ADBL), and total dendrite branching length (TDBL) were done with GraphPad Prism 6. The projection images were traced with NIH Image J, and dendritic complexity was analyzed with Image J by using the Sholl analysis plugin (Anirvan Ghosh Lab). A series of concentric circles at 15 and 25 μm intervals were drawn around the soma, and crossings of dendrites with each circle were counted automatically. Images for axon and dendrite analysis were taken by using 20× objectives under Zeiss microscope with a CCD camera.

### RNA Extraction, RT-PCR, and Real-Time PCR

The total RNAs from mouse brain tissues were isolated with TRIzol (Ambion). For gene expression, 1 μg of total RNAs were used to do reverse transcription with High-Capacity cDNA Reverse Transcription Kits (Applied Biosystems). For microRNA detection, 100 ng of total RNAs were reverse transcribed by using M-MuLV Reverse Transcriptase (New England Biolabs) and a RT primer for adding an anchored poly (T) tag to the miRNA sequence (Alvarez and Nourbakhsh, 2014). Then, the following PCR could be performed with a universal primer and a microRNA-specific primer. The PCR products were detected with SYBR Green dye, and SYBR assays were performed on an Applied Biosystems 7500 Real-Time PCR system. Relative expression was calculated by delta threshold cycles (Ct), using normalization to GAPDH for gene expression detection and to U6 for microRNA detection. The expression measures were recorded from triplicate samples, each of which was analyzed by triplicate qPCR assay. In our results, the average Cts of GAPDH, BCAT2, U6, and miR-182 are 23.64967, 25.017, 17.004, and 21.24467. The primer information for Real-Time PCR was described in **Table 1**.

**Table 1:** Primer list.

mu-miR-138-5p Forward	AGCTGGTGTTGTGAATCAG
mmu-miR-138-5p Reverse	CAGTTTTTTTTTTTTTTTCGGCCT
mmu-miR-219a-5p Forward	CAGTGATTGTCCAAACGCA
mmu-miR-219a-5p Reverse	GGTCCAGTTTTTTTTTTTTTTTAGAATTG
mmu-miR-338-3p Forward	GCAGTCCAGCATCAGTGA
mmu-miR-338-3p Reverse	GTCCAGTTTTTTTTTTTTTTTCAACA
mmu-miR-21-5p Forward	GCAGTAGCTTATCAGACTGATG
mmu-miR-21-5p Reverse	GGTCCAGTTTTTTTTTTTTTTTCAAC
mmu-miR-219b-5p Forward	CAGAGATGTCCAGCCACA
mmu-miR-219b-5p Reverse	GTCCAGTTTTTTTTTTTTTTTCGAG
mmu-miR-32-5p Forward	CGCAGTATTGCACATTACTAAG
mmu-miR-32-5p Reverse	TCCAGTTTTTTTTTTTTTTTGCAAC
mmu-miR-20a-5p Forward	GCAGTAAAGTGCTTATAGTGCAG
mmu-miR-20a-5p Reverse	GTCCAGTTTTTTTTTTTTTTTCTACCT
mmu-miR-542-3p Forward	CGCAGTGTGACAGATTG
mmu-miR-542-3p Reverse	GGTCCAGTTTTTTTTTTTTTTTCAG
mmu-miR-32-5p Forward	CGCAGTATTGCACATTACTAAG
mmu-miR-32-5p Reverse	TCCAGTTTTTTTTTTTTTTTGCAAC
mmu-miR-17-3p Forward	TGCAGTGAAGGCACTTG
mmu-miR-17-3p Reverse	GGTCCAGTTTTTTTTTTTTTTTCTACA
mmu-miR-17-3p Forward	TGCAGTGAAGGCACTTG
mmu-miR-17-3p Reverse	GGTCCAGTTTTTTTTTTTTTTTCTACA
mmu-miR-342-5p Forward	AGAGGGGTGCTATCTGTG
mmu-miR-342-5p Reverse	TCCAGTTTTTTTTTTTTTTTCAATCAC
mmu-miR-193a-3p Forward	CAGAACTGGCCTACAAAGTC
mmu-miR-193a-3p Reverse	CCAGTTTTTTTTTTTTTTTACTGGGA
mmu-miR-130a-3p Forward	CAGCAGTGCAATGTTAAAAGG
mmu-miR-130a-3p Reverse	GGTCCAGTTTTTTTTTTTTTTTATGC
mmu-miR-181d-5p Forward	AGAACATTCATTGTTGTCGGT
mmu-miR-181d-5p Reverse	GTCCAGTTTTTTTTTTTTTTTACCCA
mmu-miR-340-3p Forward	GCAGTCCGTCTCAGTTAC
mmu-miR-340-3p Reverse	GGTCCAGTTTTTTTTTTTTTTTGCT
m*Meis2* Forward	GTTTTCTTGACTGGGCTTTCCC
m*Meis2* Reverse	GGTCTCCGTACATGGAAGCG
m*Adam23* Forward	CCTAGCGCCACCAATCTCAT
m*Adam23* Reverse	AAGGTGGCATTCCTCCAGTG
m*Zdhhc8* Forward	GATTTCCTCCACTGCCGCT
m*Zdhhc8* Reverse	AGCTCTTGTCAACCATGGGC
m*Sema5a* Forward	CATCTGGGCTGGTGTGTGAC
m*Sema5a* Reverse	TCTGGGAACGTGTCTTCTGC
m*Cttn* Forward	GTGGGCCATGAGTACCAGTC
m*Cttn* Reverse	TTGCCACCGAAGCCACTAGA
m*Adcy6* Forward	TGACTGGCTGGAGGGATACA
m*Adcy6* Reverse	CCCCAAGCTGTTTTCCGTTC
m*Ntng1* Forward	CTCCGGATATTACCTGTGGA
m*Ntng1* Reverse	CTAGGATCATTTGGTCTGGA
m*Frs2* Forward	ATGCCCAGACGTAATGGTGA
m*Frs2* Reverse	TCAGAAGACCATGTGAGCACT
m*Rapgef5* Forward	GCCATTGGAGTCTGGCAGCT
m*Rapgef5* Reverse	CACAGATGCCAGCTCTTGCA
m*Rgs17* Forward	GAGAGCATCCAGGTCCTAGA
m*Rgs17* Reverse	CTCGTATATCATCCTGGCCT
m*Pdzd8* Forward	CACACCCTGCCCAGTTACAA
m*Pdzd8* Reverse	CCACTGCTCAGCTCAAGTGT
m*Rcor1* Forward	GCTCGCTGGACAACGGAAGA
m*Rcor1* Reverse	CAGGTCCATTGGTCTCGTCT
m*Pax8* Forward	GTTTGAGCGGCAGCATTACC
m*Pax8* Reverse	TCCTGCTTTATGGCGAAGGG
m*Bcl11a* Forward	CATCTTCCCTGCGCCATCTTT
m*Bcl11a* Reverse	GAATGGCTTCAAGAGGTTCGG
m*Bcat2* Forward	GAAGACGGAGTACTGGAGCTG
m*Bcat2* Reverse	TGGCTCCGTACTGAATAGCC
m*Bcact2* 3′ UTR to pmirGLO Forward	CGAGCTCAGAGACTCAAGAGAAGTGCAATAGG
m*Bcact2* 3′ UTR to pmirGLO Reverse	TGCTCTAGATTCTTGTTCTTTGGCATTTTATTTC

### Immunostaining

Neural stem cells were cultured for 2 days in StemPro NSCs culture medium (Gibco). The NSCs and differentiated neurons were collected, washed three times with PBS, fixed in 4% PFA for 20 min at room temperature, and permeabilised with 0.3% Triton X-100 for 10 min. After blocked with 5% BSA for 10 min, cells were incubated with primary antibodies overnight at 4°C, and signals were detected by a fluorescence microscope. Cell nuclei were visualized with DAPI. Primary antibodies included: rabbit anti-Nestin (Santa Cruz Biotechnology #sc-20978) and rabbit anti-β III tubulin (Abcam #ab18207); secondary antibodies included a donkey anti-rabbit IgG secondary antibody (Life Technologies #A16036).

### Construction of Vectors

3′ UTR-containing fragments of *BCAT2* were amplified by PCR using Platinum Pfx DNA Polymerase and inserted into the SacI and XbaI sites of pmir-GLO (Promega). The primer information was described in **Table 1**.

### Luciferase Reporter Assay

HEK-293T cells were plated in 96-well plates at 2.5 × 10^4^ cells per well and co-transfected with 60 nM scramble mimics or 60 nM miR-182 mimics plus 0.5 ng/μl plasmids of pmir-GLO-mBCAT2-3′ UTR using Lipofectamine 3000 (Life Technologies), and each group is triplicate. 293T cells were cultured for 2 days after transfection and lysed with Dual-Glo Luciferase reagent. Luciferase activity was detected with the Dual-Glo Luciferase Assay System (Promega) according to the manufacturer’s protocol, and the results were determined by normalizing to the Renilla luciferase activities of three independent experiments.

### Cell Proliferation Assay

We used PI (Sigma) /Hoechst (Life Technologies #H1399) and Trypan Blue to detect cell apoptosis, and used a kit (Promega, G3580) to detect cell proliferation ability. For the cell proliferation assay kit, cortical neurons were plated in 4 × 10^4^ cells per well in 96-well plate, and transfected with microRNAs at 1 DIV. Pipet 20 μl of one solution reagent into each well of 96-well plate containing 100 μl of culture medium. Incubate the plate at 37°C for 1–4 h, and then record the absorbance at 490 nm.

### Ingenuity Pathway Analysis (IPA)

Datasets represented a gene list were submitted to the Ingenuity Pathway Analysis Tool (IPA tool; Ingenuity H Systems, Redwood City, CA, USA^1^) for core analysis.

### Statistical Analysis

The results presented are the average of at least three experiments with standard errors of the mean. Statistical analyses were performed with an unpaired, two-tailed Student’s *t*-test and one-way ANOVA, and the data remain normally distributed. One-way ANOVA with *post hoc* test was performed for multiple comparisons. A *p*-value of <0.05 was considered to indicate statistical significance. Statistical analyses were performed using GraphPad Prism 6.

## Results

### MiR-182 is Enriched in Neurons

**FIGURE 1:**
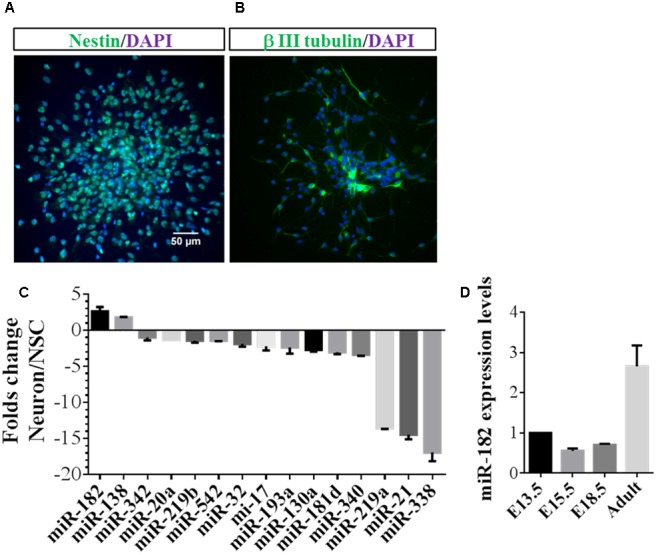
**MiR-182 is highly expressed in neurons compared with neural stem cells. (A)** Undifferentiated neural stem cells which were dissociated from adult mouse brain expressed Nestin. **(B)** Neurons differentiated from neural stem cells expressed β III tubulin. **(C)** Identification of specific miRNAs in neurons compared with neural stem cells. The relative ratios comparing differentiated to undifferentiated NSCs are shown. MiR-182 and miR-138 showed specific high expression in neurons compared with undifferentiated neural stem cells. **(D)** MiR-182 expression during mouse brain development at embryonic days 13.5, 15.5, 18.5, and adult mouse.

To identify microRNAs that may regulate neuronal development, NSCs were dissociated from the SVZ zone of adult mouse brain and differentiated into neurons (Gage, 2000). We stained the NSCs with primary antibody of anti-Nestin (**Figure 1A**) and stained the neurons differentiated from NSCs with β III tubulin (**Figure 1B**). Recent evidence shows that numbers of microRNAs are specifically expressed in neurons and promote axon extension (Jovicic et al., 2013; Hancock et al., 2014). In a companion paper, we designed primers and quantitatively identified microRNAs that were enriched in neurons relative to NSCs. Several microRNAs, particularly miR-182 and miR-138, showed enrichment in neurons but not in NSCs, whereas other microRNAs, such as miR-338, miR-21, and miR-219a were enriched in NSCs but not in neurons (**Figure 1C**). These results were consistent with previous results revealing that miR-182 is enriched in neurons compared with astrocytes (Smrt et al., 2010), and miRNAs specifically enriched in neurons were identified by microRNA array (Jovicic et al., 2013).

MiR-182 is known to be highly expressed in sensory cell types (Li et al., 2010) and involved in plasticity and memory (Griggs et al., 2013). We reasoned that if miR-182 were critical for neurodevelopment and function, it should be expressed in neurons of mouse brain. Hence, we detected miR-182 expression levels during different stages of mouse brain development and found that they increased significantly from E13.5 to adult (**Figure 1D**). We hypothesized whether miR-182 has functions in the later stage of neurite growth, and found that the expression of miR-182 was increased from 2 days after birth to adult (**Supplementary Figure S1A**). *In vitro* results showed that miR-182 expression levels were upregulated from 1 to 7 DIV in primary cultured neurons (**Supplementary Figure S1B**). Together, these data and other published literatures suggest that miR-182 plays functional roles in neurons.

### MiR-182 Promotes Axon Outgrowth in Cortical Neurons

**FIGURE 2:**
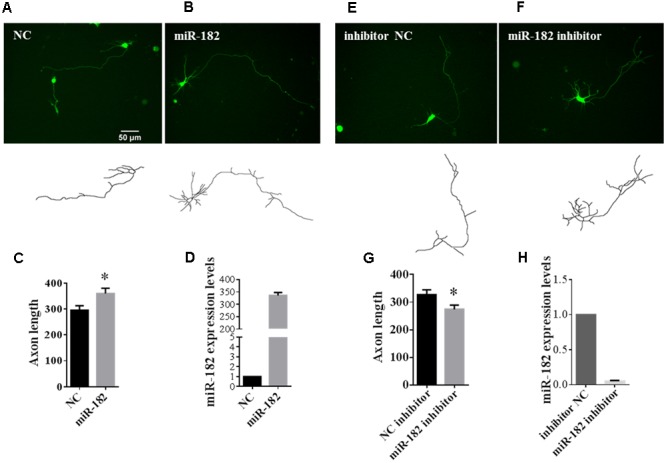
**MiR-182 promotes axon outgrowth. (A,B)** A schematic diagram showing scramble microRNA and miR-182 mimics plus *GFP*-encoding plasmid that were transfected into cortical neurons at 1 DIV and imaged at 3 DIV. **(C)** Quantification of axon length. Data were presented as mean ± SEM. ^∗^*p* < 0.05 by Student’s *t*-test, *N* = 3 independent experiments; at least 35 neurons were analyzed in each experiment. **(D)** The expression levels of miR-182 were measured by qRT-PCR. **(E,F)** Blocking of endogenous miR-182 reduced axon length compared with controls. **(G)** Quantification of axon length. Data were presented as mean ± SEM. ^∗^*p* < 0.05 by Student’s *t*-test, *N* = 3 independent experiments; at least 35 neurons were analyzed in each experiment. **(H)** Expression levels of miR-182 as measured by qRT-PCR.

MicroRNAs were found to play important roles in promoting neuronal differentiation and maturation. Here, we tried to investigate the function of miRNAs in neuronal maturation. As miR-182 is enriched in neurons, we reasoned that miR-182 might regulate neuron development during brain development. We co-transfected with miR-182, miR-138, and miR-31 each plus *GFP*-encoding plasmid into primary cultured cortical neurons at 1 DIV to investigate whether microRNAs overexpression in neurons could affect axon outgrowth. At 36 h after transfection, individual cortical neurons expressing GFP were imaged using fluorescence microscopy. The morphology of axons and soma, which was manually traced and measured by Image J software, showed that miR-182 promoted axon outgrowth by increasing axon length (**Figures 2A,B**). The statistical results are described in **Figure 2C**. In contrast, miR-138 showed no difference in axon outgrowth (data not shown). The overexpression of miR-182 was confirmed by qRT-PCR (**Figure 2D**). To verify the function of miR182 in regulating axon outgrowth, we blocked the endogenous miR-182 by transfecting with antisense oligonucleotides (**Figures 2E,F**). The results showed that miR-182 inhibitor could suppress axon outgrowth compared with the control group (**Figure 2G**), and miR-182 expression was detected by quantitative real-time PCR (**Figure 2H**).

**FIGURE 3:**
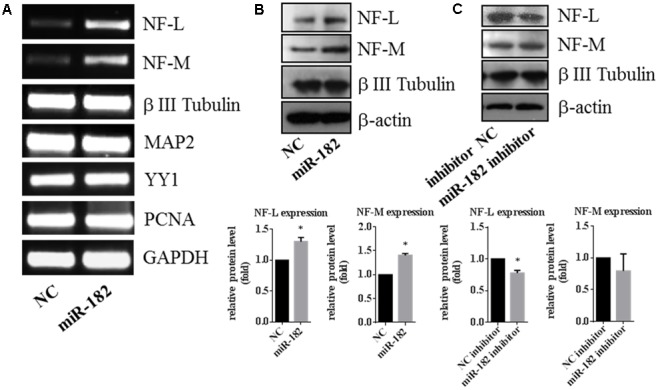
**Expression of neurofilament is regulated by miR-182. (A)** Expressions of neurofilament-M and -L were upregulated by miR-182 in the RT-PCR results, whereas the reference genes (MAP2 and β III Tubulin) showed no differences. **(B)** MiR-182 promoted neurofilament-M and -L expression by western blot (^∗^*p* < 0.05). **(C)** Blocking of the endogenous miR-182 inhibited the expression of neurofilament-L in protein level, and had no effects on neurofilament-M (^∗^*p* < 0.05).

As a neuron marker, neurofilaments (NFs), which consist of three subunits termed neurofilament-L (NF-L), neurofilament-M (NF-M) and neurofilament-H, are thought to provide structural support for mature axons (Nixon and Shea, 1992; Lee and Shea, 2014). We found that the expression of NF-L and NF-M was regulated by miR-182. Overexpression of miR-182 increased the expression of NF-L and NF-M in mRNA levels by RT-PCR, but β III tubulin and MAP2 as reference genes were not influenced by miR-182 (**Figure 3A**); in protein levels by western blot (**Figure 3B**). Western blot results showed that inhibition of miR-182 decreased the protein level of NF-L, but it had no influence on the protein levels of NF-M by western blot (**Figure 3C**). It indicated that miR-182 promoted axon outgrowth by regulating NF-L.

We tested the cell viability by PI/Hoechst staining and Trypan blue staining after transfection with microRNAs (**Supplementary Figure S2A**), and detected the cell dynamic viability after transfection for 3 days by cell proliferation assay (**Supplementary Figure S2B**). **Supplementary Figure S2C** showed the transfection efficiency of scramble mimics which conjugated with FAM fluorescence dye.

### MiR-182 Regulates Dendrite Branching Out

**FIGURE 4:**
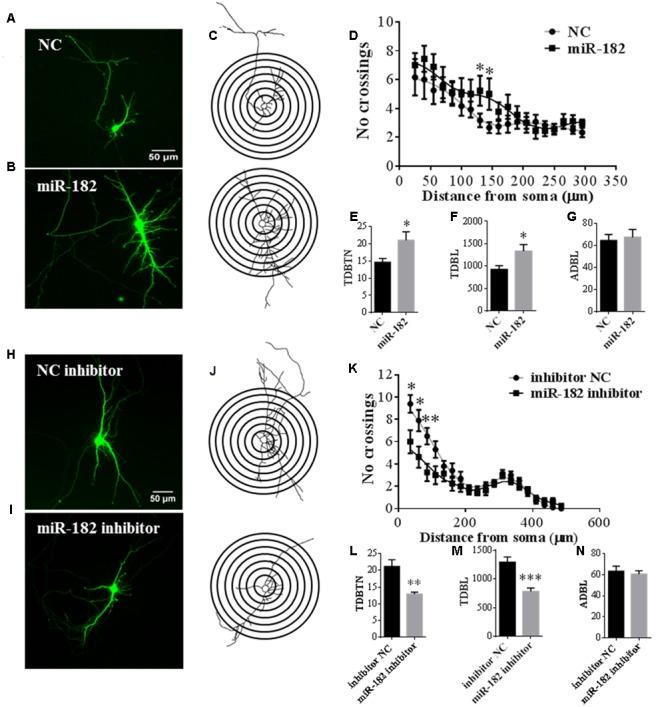
**MiR-182 promotes dendrite branching out. (A,B)** Cortical neurons were transfected with scramble mimics and miR-182 mimics (60 nM) at 5 DIV. After 48 h, neurons were harvested and images were recorded. A representative image is shown each for neurons transfected with scramble microRNA mimics and miR-182 mimics. **(C)** Representative picture of the Sholl analysis. **(D)** Quantitative results of the number of dendrite process intersections by Sholl analysis. MiR-182 increased dendritic branching at the distance of 130 and 145 μm from the soma. One-way ANOVA, Tukey’s post-test (^∗^*p* < 0.05); 35 neurons were analyzed in each condition. **(E–G)** Quantification of total dendritic branch tip number (TDBTN), average dendritic branch length (ADBL) and total dendritic branch length (TDBL) in cortical neurons at 7 DIV (^∗^*p* < 0.05). **(H,I)** Cortical neurons at 5 DIV were transfected with mimics of inhibitor negative control and miR-182 inhibitor (60 nM) plus *GFP*-encoding plasmid. Blockage of endogenous miR-182 decreased dendrite complexity, as determined by Sholl analysis. **(J)** Representative images of neurons subjected to Sholl analysis. **(K)** Quantification of dendrite complexity by Sholl analysis showed that blockage of endogenous miR-182 decreased dendrite branching at the distances of 35, 60, and 85 μm from the soma (^∗^*p* < 0.05, ^∗∗^*p* < 0.01). **(L–N)** Quantification of total dendritic branch tip number (TDBTN), average dendritic branch length (ADBL) and total dendritic branch length (TDBL) for dendrites (^∗∗^*p* < 0.01, ^∗∗∗^*p* < 0.001).

Dendrites have important functional implications in synapse formation and electroneurographic signal-passing in mature neurons (Jan and Jan, 2010). As miR-182 is known to be enriched in neurons, we further studied the functions of miR-182 on dendrite development and neuron maturation. To evaluate its influence on the complexity of dendrite tree, primary cortical neurons were co-transfected with *GFP*-encoding plasmid containing miR-182 mimics and scramble mimics at 5 DIV. Ectopic expression of miR-182 significantly increased the dendrite complexity (**Figures 4A,B**). We performed Sholl analysis to analyze the dendrite morphology by measuring the number of dendrites that intersected concentric circles at different distances from the soma (Sholl, 1953) (**Figure 4C**). The results showed that miR-182 significantly increased the complexity of the dendrite tree at a distance between 130 and 145 μm from the soma (**Figure 4D**). TDBTN and TDBL were significantly increased, but ADBL remained unchanged (**Figures 4E–G**).

In contrast, the inhibition of miR-182 expression resulted in a significant reduction of total dendritic length and branch numbers (**Figures 4H,I**); and the number of intersections was significantly reduced between the distance of 35–85 μm from the soma (**Figures 4J,K**); TDBTN and TDBL were significantly reduced (**Figures 4L,M**), but ADBL was not changed (**Figure 4N**). Together, these data suggested that miR-182 overexpression caused changes in dendrite branching and neuronal maturation; thus, it might play important roles during mouse brain development.

### PTEN/AKT Pathway is Involved in Regulating Dendrite Branching Out

**FIGURE 5:**
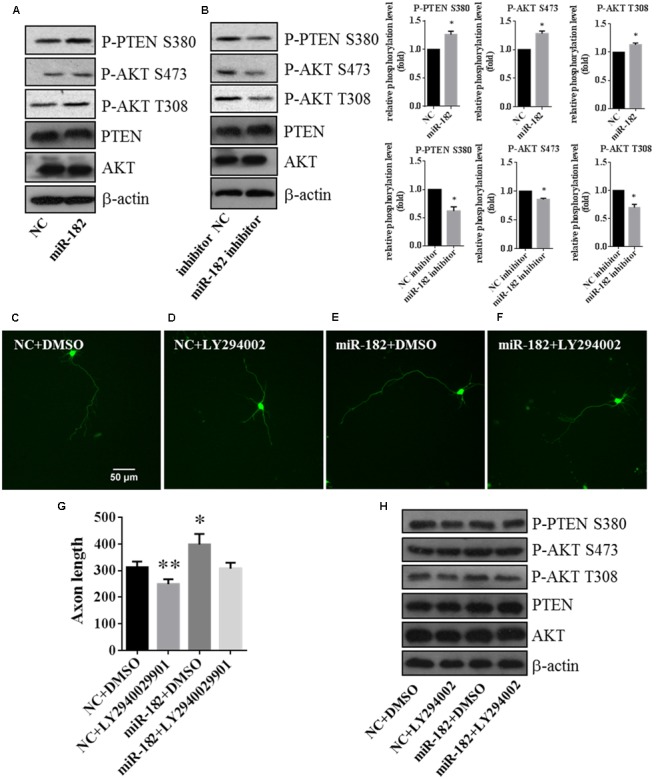
**PTEN/AKT pathway is involved in regulating axon outgrowth. (A)** Cellular fractions overexpressing miR-182 were analyzed by western blot using antibodies against P-AKT S473, P-AKT T308, AKT, PTEN, and P-PTEN S380. β-actin was used as the loading control, and the data represented the results of at least three different experiments. **(B)** Phosphorylation of AKT and PTEN was analyzed when miR-182 was downregulated. **(C–F)** Representative cortical neurons transfected with microRNA scramble plus DMSO, microRNA scramble plus LY294002, miR-182 plus DMSO and miR-182 plus LY294002. **(G)** Quantification of axon length (^∗^*p* < 0.05, ^∗∗^*p* < 0.01). **(H)** Primary cultured cortical neurons were transfected with scramble microRNA plus DMSO, microRNA scramble plus LY294002, miR-182 plus DMSO and miR-182 plus LY294002, and analyzed by western blot using antibodies against P-AKT S473, P-AKT T308, AKT, PTEN, and P-PTEN S380.

We investigated the mechanism of miR-182 in the regulation of neuronal development, and detected the activity of the PI3K/AKT, RAF/ERK, and JNK pathways. MicroRNAs regulate dendrite branching out via AKT pathway, which is known to play roles in the regulation of dendritic morphogenesis and to increase the complexity of the dendritic tree (Kumar et al., 2005; Xue et al., 2016). We found that overexpression of miR-182 increased the activity of AKT in T308 and S473 and blockage of the endogenous miR-182 inhibited AKT activity (**Figures 5A,B**), but there are no effects on Erk activity by miR-182 (**Supplementary Figure S3**). PTEN is an inhibitor of PI3K which promotes AKT activity, and the phosphorylation of PTEN S380 negatively regulates PTEN activity by preventing its recruitment into a protein complex (Vazquez et al., 2001). In our results, overexpression of miR-182 inhibited the activity of PTEN by increasing its phosphorylation (**Figure 5A**), and the phosphorylation of PTEN was decreased when the endogenous miR-182 was blocked (**Figure 5B**). Activation of the PI3K/AKT pathway increases dendritic complexity in neurons (Kumar et al., 2005), consistent with our findings of the induction of axon outgrowth and dendritic branching by miR-182.

Meanwhile, to investigate whether endogenous AKT activities are necessary for axon outgrowth, we inhibited the PI3K/AKT pathway by adding PI3K inhibitor LY294002 plus transfection with scramble mimics and miR-182 mimics. We found that the AKT activity (S473 and T308) and the phosphorylation of PTEN (S380) decreased compared with the non-drug group (**Figure 5H**). The statistic of axon length was consistent with the western blot result that LY294002 could rescue the influence on AKT activity regulated by miR-182 (**Figures 5C–G**). These data verified that miR-182 promoted neuronal maturation by activating the AKT pathway.

Taken together, these results showed that miR-182 inhibited the activity of PTEN and promoted the phosphorylation of AKT, indicating that miR-182 regulated axon outgrowth and dendrite branching out involving the PTEN/AKT pathway.

### *BCAT2* is a Translational Target of miR-182

**FIGURE 6:**
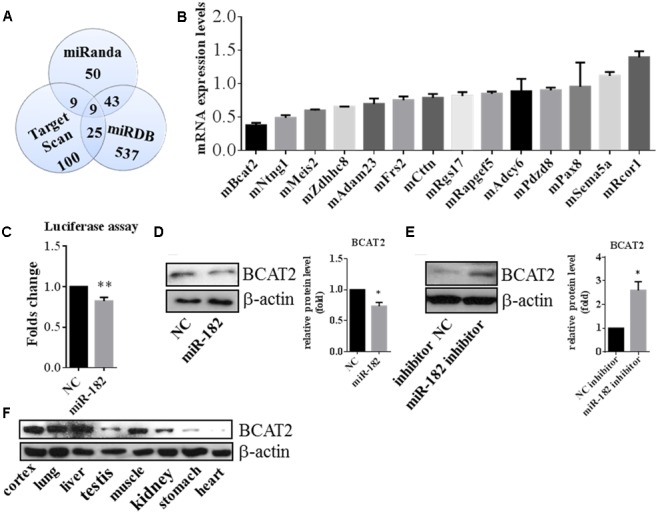
**BCAT2 is a translational target of miR-182. (A)** Predicting targets of miR-182-5p by comparing the performance of three databases, including Miranda, Target Scan, and miRDB. **(B)** Quantitative real-time PCR indicated that BCAT2 expression was significantly decreased in cortical neurons by miR-182 mimics. **(C)** HEK-293T cells were transfected with miR-182 mimics and scramble mimics each plus luciferase-*BCAT2*-3′ UTR reporter vector. MiR-182 reduced the luciferase intensity compared with the control (^∗∗^*p* < 0.01). **(D)** MiR-182 reduced the expression of BCAT2 in protein levels in primary cultured neurons (^∗^*p* < 0.05). **(E)** Blockage of endogenous miR-182 increased the expression of BCAT2 by western blot in primary cultured neurons (^∗^*p* < 0.05). **(F)** Expression pattern of BCAT2 in different tissue of mouse embryo by western blot.

To explore the mechanism of miR-182 in regulation of neurite outgrowth, we attempted to identify the target gene of miR-182. First, we predicted the miR-182 target genes using three public databases, including miRanda, Target Scan, and miRDB; and identified nine candidate targets to be common (**Figure 6A**). We compared the expression of 14 target candidates of miR-182 by quantitative real-time PCR in primary cultured cortical neurons, which were transfected with scramble mimics and miR-182 mimics at 1 DIV and lysed at 3 DIV. We found that *BCAT2* expression was significantly downregulated by miR-182 (**Figure 6B**). *BCAT2* is known to be a target of miR-182 in PIGF mice and mouse embryonic fibroblasts (Li et al., 2016). Furthermore, a 3′ UTR-containing fragment of mouse *BCAT2* was amplified and cloned into the luciferase reporter plasmid to detect the luciferase activity. The luciferase activity was significantly downregulated by miR-182 (**Figure 6C**), consistent with the results of a previous report (Li et al., 2016). Meanwhile, protein levels of BCAT2 were significantly decreased in cortical neurons overexpressing miR-182 (**Figure 6D**), but the expression of BCAT2 increased when blocking the endogenous miR-182 by the inhibitor (**Figure 6E**). Meanwhile, we detected the BCAT2 protein expression in different tissues of the E15.5 mouse; found that BCAT2 was highly expressed in cortex, lung, and liver (**Figure 6F**).

**FIGURE 7:**
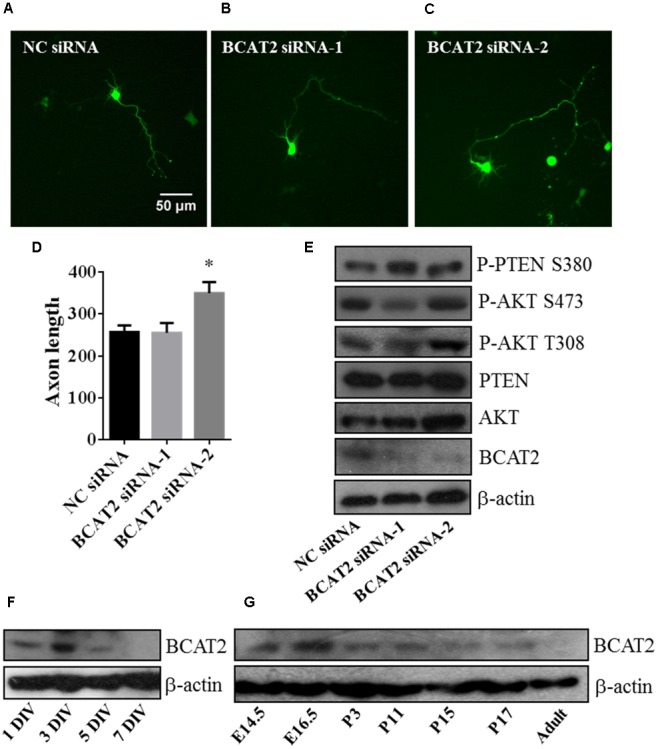
**Blockage of endogenous BCAT2 promotes axon outgrowth and increases AKT activity. (A–C)** Cortical neurons were transfected with negative control siRNA, BCAT2 siRNA-1, and BCAT2 siRNA-2 each plus *GFP*-encoding plasmid at 1 DIV. **(D)** Quantification of axon length and BCAT2 siRNA-2 increased axon length (^∗^*p* < 0.05). **(E)** BCAT2 siRNA-2 increased the phosphorylation of AKT S473, T308, and PTEN S380 in primary cultured neurons. **(F)** BCAT2 expressions in cultured neurons at 1, 3, 5, and 7 DIV. **(G)** BCAT2 expressions in mouse brain cortex from embryonic 14.5 to adult.

To verify the function of BCAT2, we transfected with two siRNA mimics into primary cultured neurons for downregulating the endogenous BCAT2, and deficiency of BCAT2 by siRNA-2 promoted axon outgrowth (**Figures 7A–D**). In western blot results, BCAT2 siRNA-2 increased the activity of AKT and decreased the activity of PTEN (**Figure 7E**). Meanwhile, we investigated the expression profile of BCAT2 in cultured neurons and brain tissue after birth, and found that the expression tendency was decreased (**Figures 7F,G**); it was consistent with the expression profile of miR-182 (**Supplementary Figure S1**). In cardiomyocytes, *BCAT2* deletion promotes AKT activity by increasing the phosphorylation of Ser-473 (Li et al., 2016). MiR-182 may regulate neurite outgrowth by targeting *BCAT2* and further increasing AKT activity and promoting neuronal maturation.

## Discussion

Literatures described that microRNAs play vital roles in neuronal development and are essential for the development of central nervous system (Wayman et al., 2008). MiR-182 is highly expressed in the retina (Xu et al., 2007) and identified as the most abundant among axonal miRNAs of RGCs. Loss of miR-182 causes RGC axon targeting defects *in vivo* (Bellon et al., 2017). In a companion paper, we compared the expression levels of several microRNAs between NSCs and neurons, and found that miR-182 was enriched in neurons. Overexpression of miR-182 promoted axon outgrowth and dendrite branching out in mouse cortical neurons; it implicated that miR-182 played a role in neuronal maturation in the early stage of neuronal development. The conclusion of a study described that miR-182 could regulate trigeminal sensory neurons by promoting dendrite branching (Wang et al., 2016), and it was consistent with our results.

The small GTPases of Rho subfamily are critical regulators of axon outgrowth and dendrite elaboration. Cdc42 (cell division cycle 42), Rac1 (Ras-related C3 botulinum toxin substrate 1), and RhoA (Ras homologous member A) are three well-studied Rho GTPases. Cdc42 and Rac1 are positive regulators of axon growth and dendrite development, but RhoA is a negative regulator (Luo, 2000; Suo et al., 2012). Recently, a study showed that miR-182 is involved in structural plasticity and memory formation by regulating the expression of cortactin and Rac1 (Griggs et al., 2013). In the mentioned literature, miR-182 is low expressed in lateral amygdala, and the inner mechanism of miR-182 regulation of amygdala-dependent memory formation was still unclear. There is a possibility that miR-182 plays distinct role in different areas of brain, like cerebral cortex and lateral amygdala.

In our results, miR-182 increased the expression of NF-L and NF-M in mRNA and protein levels. It indicates that miR-182 regulates neurites outgrowth through indirect effects in NF-L and NF-M. MiR-182 may bind to other target which is the repressor of neurofilaments, and eliminate the effects of inhibition for the neurofilaments expression. Because miR-182 has many other potential targets predicted by the software, whether miR-182 regulates neurite outgrowth through neurofilaments on other targets needs to be investigated in the future.

MiR-182 inhibits apoptosis and promotes survival in medulloblastoma cells by regulating the PI3K/AKT/mTOR signaling axis (Weeraratne et al., 2012). In our work, miR-182 promoted neuronal maturation by increasing AKT phosphorylation and inhibiting PTEN activity. The PTEN/AKT pathway is critical for dendritic morphogenesis (Kumar et al., 2005) and involved in neuron survival controlled by microRNAs (Wong et al., 2013; Han et al., 2014). BCAT2 is expressed in brain tissue (Hull et al., 2012; Zampieri et al., 2013), but no evidence was offered for the function of BCAT in neurite growth before. In this paper, we presented the first report to introduce BCAT’s effects in neurite outgrowth, and discovered that *BCAT2* could be considered as a target of miR-182 for regulating neurite outgrowth. Blockage of the endogenous BCAT2 by siRNA promoted axon outgrowth through PTEN/AKT pathway. The results are partly consistent with a previous report that *BCAT2* is a target of miR-182, and BCAT2 deficiency promotes AKT activation by increasing the phosphorylation of Ser473 in cardiomyocytes (Li et al., 2016). BCAT2 catalyzes the first step in the mitochondrial catabolism of BCAAs, and BCAAs provide nitrogen for the synthesis of glutamate, an excitatory neurotransmitter; BCAAs appear to increase the phosphorylation of AKT S473 by activating mTORC2 (Tato et al., 2011; Li et al., 2016). BCAAs catalyzed by BCAT2 may be the direct regulator of AKT and PTEN, but we have no evidence.

Inhibition of BCAT may be useful for the treatment of behavioral and neurodegenerative disorders (Hu et al., 2006). As the expression of BCAT2 was decreased after birth (**Figure 7G**), BCAT2 expression pattern may be different in neuron injury. We chose several published target genes of miR-182 and PTEN/AKT pathway to do Ingenuity Pathway Analysis (IPA) and found it was more related to cell morphology and nervous system development (**Supplementary Figures S4A,B**). MiR-182 plays important roles in the synaptic connectivity of photoreceptors and retinal regeneration (Lumayag et al., 2013), and a literature described that miR-182 plays a role in regulating CLOCK expression after hypoxia-ischemia brain injury (Ding et al., 2015). It is worthy of further investigation for the function of miR-182 and BCAT2 in neuron regeneration.

## Conclusion

**FIGURE 8:**
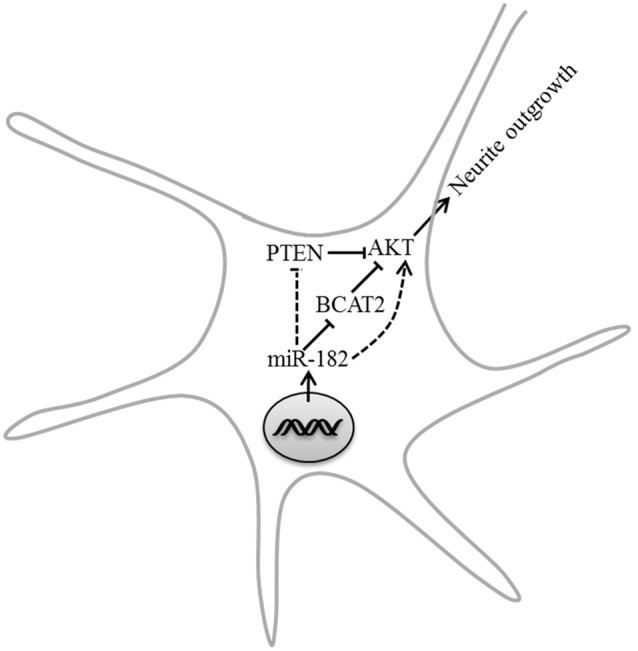
**Model depicting the roles of miR-182 in regulating neurite outgrowth.** MiR-182 was involved in the inhibition of BCAT2 and resulted in increasing AKT activity, which might lead to neurite outgrowth.

Our results first show that one of neuron-enriched microRNAs, miR-182, has an important modulatory role in neuron development. Both overexpression and inhibition of miR-182 have significant but opposite effects in axon outgrowth and dendrite branching out, and PTEN/AKT pathway is involved in the regulation of neurite outgrowth by miR-182. We also find that BCAT2 is a target of miR-182; deficiency of BCAT2 increases the activity of AKT and promotes neurite growth (**Figure 8**).

## Author Contributions

Conceived and designed the experiments: WW, GL, and WP. Performed the experiments and analyzed the data: WW, GL, XS, HL, and WP. Wrote the paper: WW, GL, and WP. All authors contributed to the revision of the article and approved the final version of the manuscript.

## Conflict of Interest Statement

The authors declare that the research was conducted in the absence of any commercial or financial relationships that could be construed as a potential conflict of interest.

## Footnotes

1 http://www.ingenuity.com

## Supplementary Material

FIGURE S1 **Expression profile of miR-182 in brain cortex after birth and cultured neurons. (A)** Expression profile of miR-182 in brain cortex from postnatal 2 to adult. **(B)** MiR-182 expression profile in cultured neurons at 1, 3, 5, and 7 DIV.

FIGURE S2 **Cell viability test. (A)** PI/Hoechst and Trypan blue staining for cultured neurons transfected with microRNA scramble, miR-182 mimics, inhibitor negative control mimics, and miR-182 inhibitor mimics. **(B)** Cell proliferation assay for cultured neurons transfected with microRNA scramble, miR-182 mimics, inhibitor negative control mimics, and miR-182 inhibitor mimics from day 1 to day 3 after transfection. **(C)** MicroRNA transfection efficiency test in neurons and FAM fluorescence dye is conjugated to microRNA.

FIGURE S3 **MiR-182 had no effect on Erk pathway.** Detecting phosphorylation of Erk1/2 by western blot, and had no effects by miR-182.

FIGURE S4 **Ingenuity Pathway Analysis (IPA) for miR-182 and PTEN/AKT pathway. (A)** The network relationship among targets genes of miR-182 and PTEN/AKT. **(B)** The influence on different physiological medicine by miR-182 and PTEN/AKT pathway in IPA.
