# Supplementary figures and images for: MicroRNA-182 Regulates Neurite Outgrowth Involving the PTEN/AKT Pathway
Source: Front Cell Neurosci. 2017 Apr 10;11:96. doi: 10.3389/fncel.2017.00096 (PMC5385363; doi:10.3389/fncel.2017.00096)

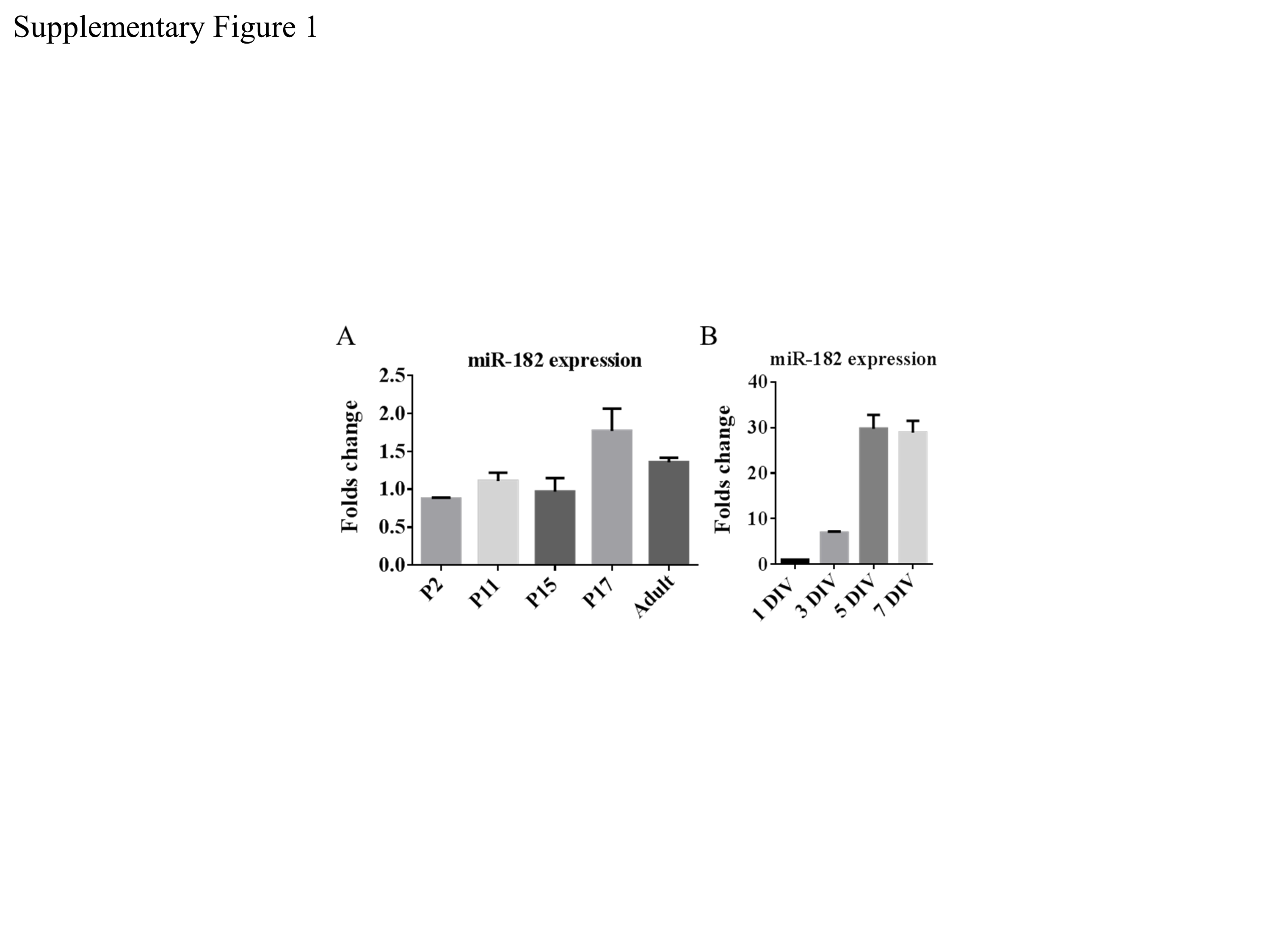

Supplement: FIGURE S1 — Expression profile of miR-182 in brain cortex after birth and cultured neurons. (A) Expression profile of miR-182 in brain cortex from postnatal 2 to adult. (B) MiR-182 expression profile in cultured neurons at 1, 3, 5, and 7 DIV. [file Image_1.TIF]

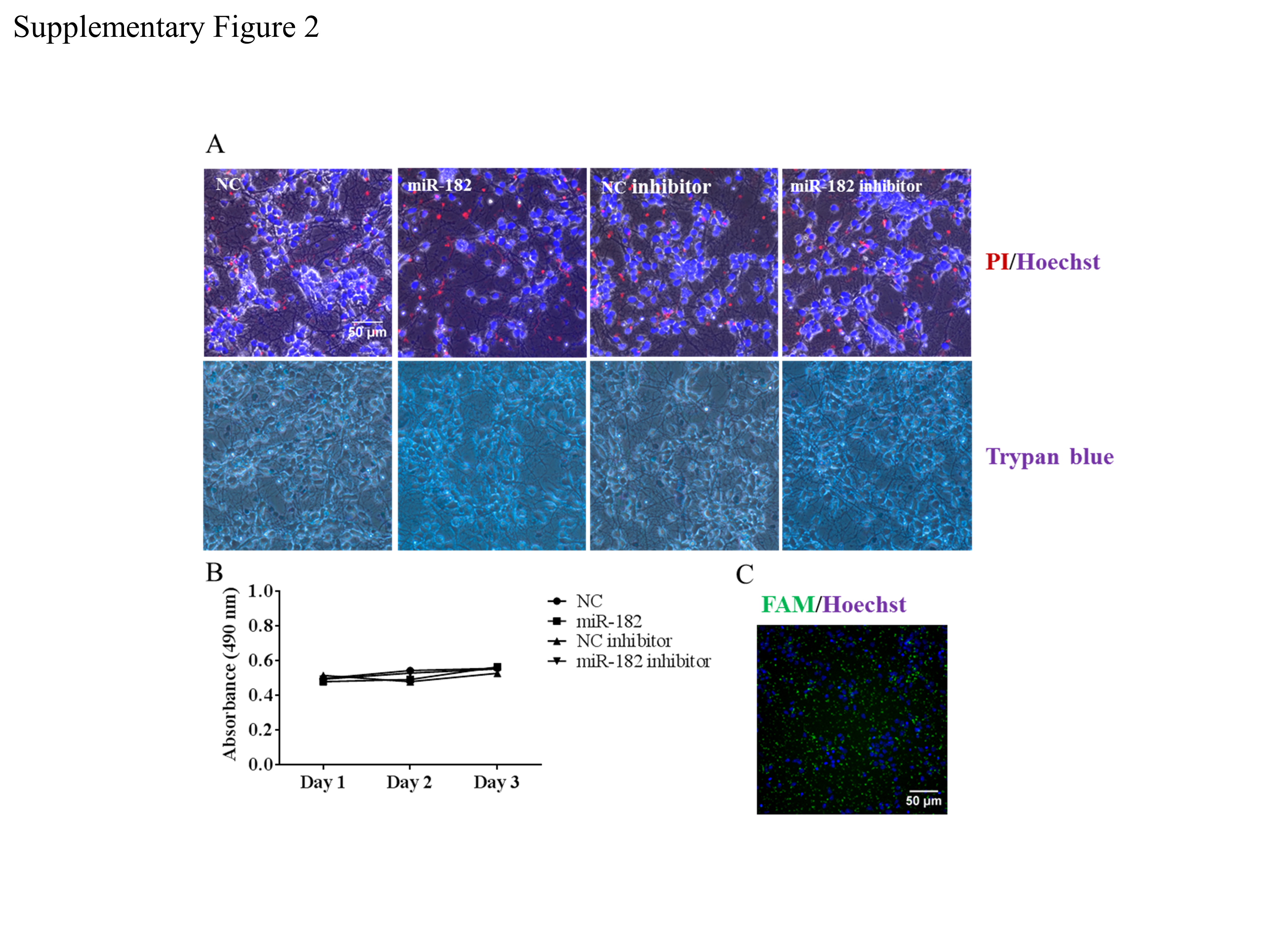

Supplement: FIGURE S2 — Cell viability test. (A) PI/Hoechst and Trypan blue staining for cultured neurons transfected with microRNA scramble, miR-182 mimics, inhibitor negative control mimics, and miR-182 inhibitor mimics. (B) Cell proliferation assay for cultured neurons transfected with microRNA scramble, miR-182 mimics, inhibitor negative control mimics, and miR-182 inhibitor mimics from day 1 to day 3 after transfection. (C) MicroRNA transfection efficiency test in neurons and FAM fluorescence dye is conjugated to microRNA. [file Image_2.TIF]

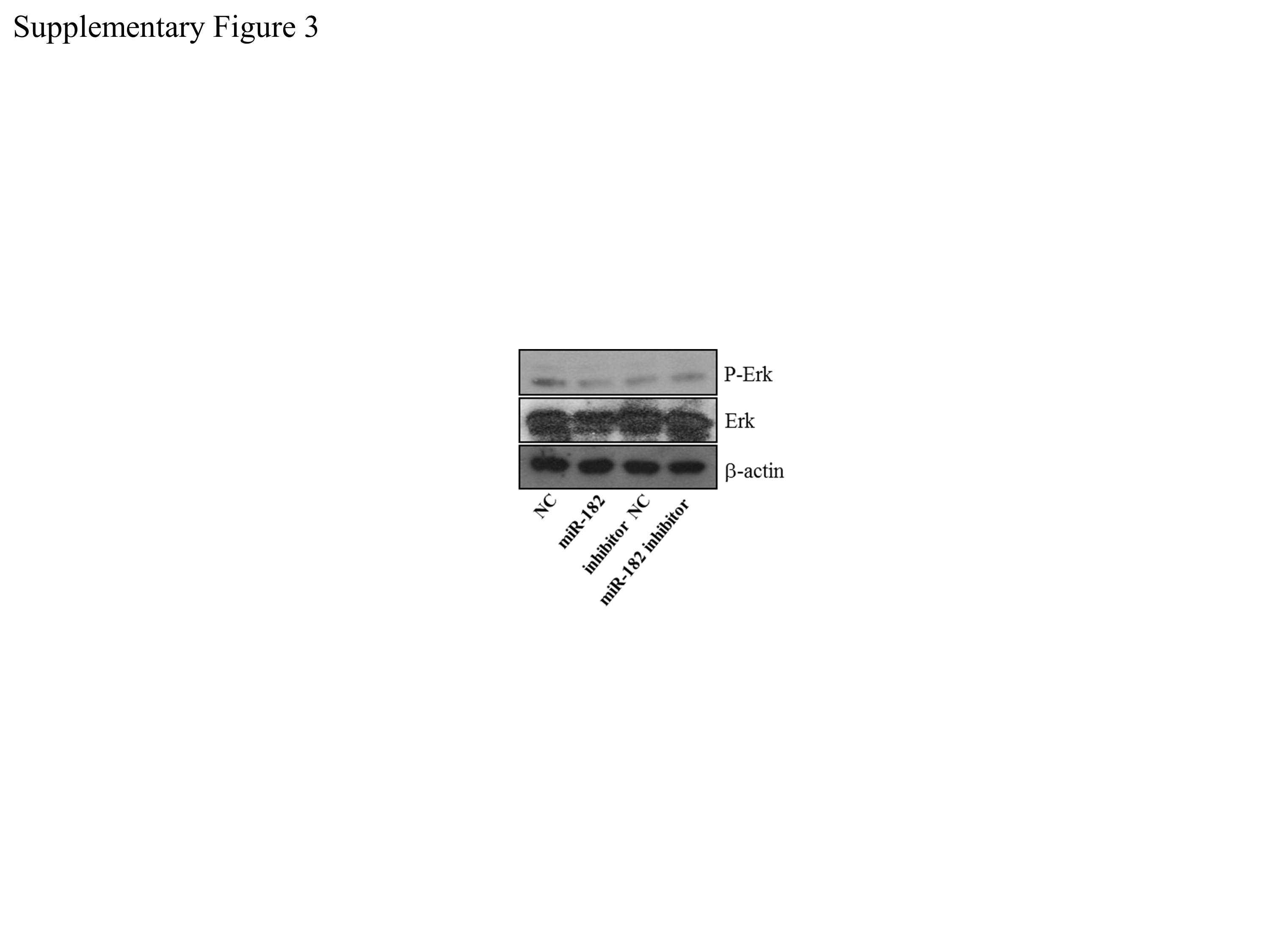

Supplement: FIGURE S3 — MiR-182 had no effect on Erk pathway. Detecting phosphorylation of Erk1/2 by western blot, and had no effects by miR-182. [file Image_3.TIF]

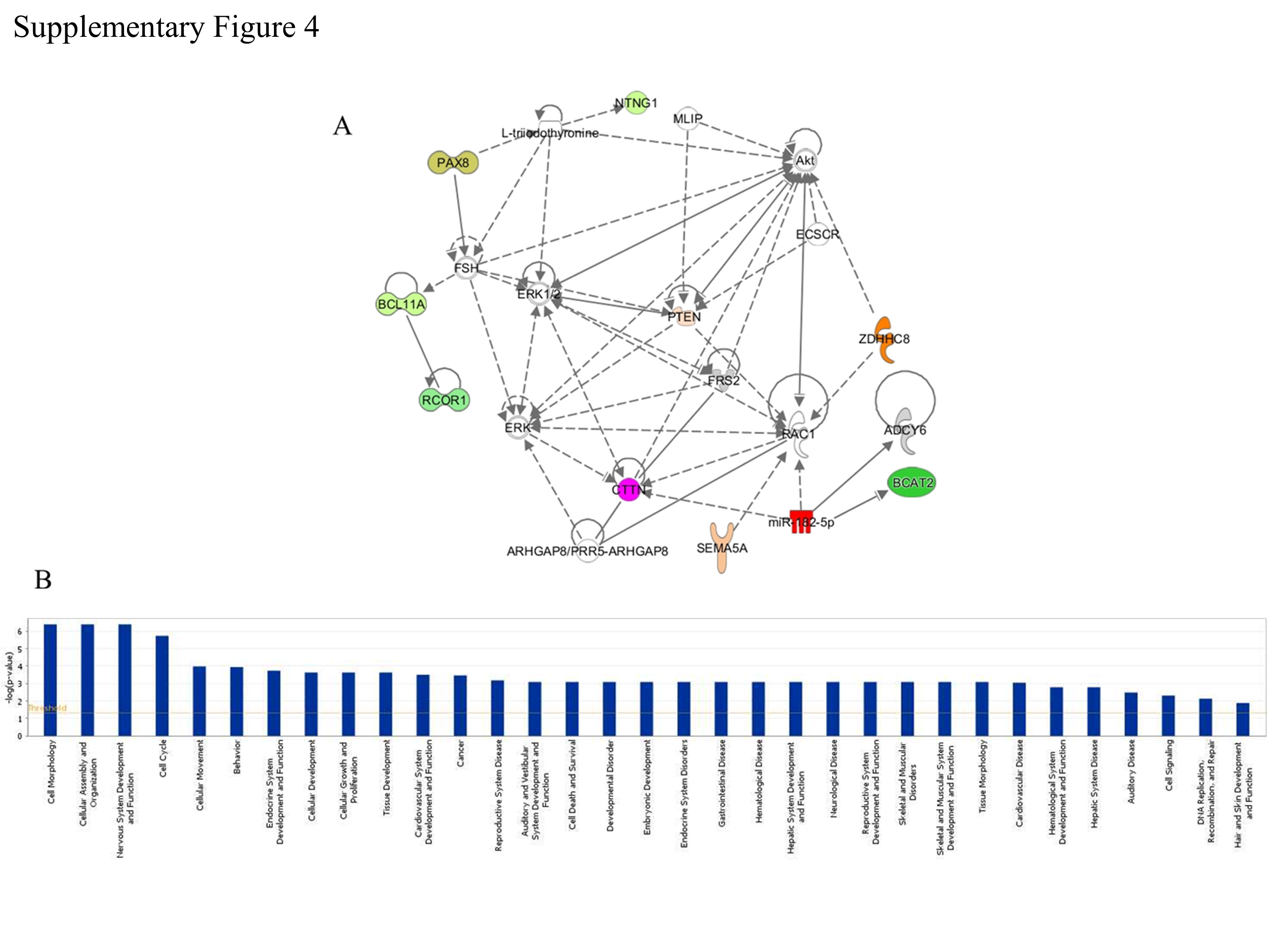

Supplement: FIGURE S4 — Ingenuity Pathway Analysis (IPA) for miR-182 and PTEN/AKT pathway. (A) The network relationship among targets genes of miR-182 and PTEN/AKT. (B) The influence on different physiological medicine by miR-182 and PTEN/AKT pathway in IPA. [file Image_4.TIF]
